# Are there differences in HIV retention in care between female and male patients in Indonesia? A multi-state analysis of a retrospective cohort study

**DOI:** 10.1371/journal.pone.0218781

**Published:** 2019-06-25

**Authors:** Annisa Rahmalia, Michael Holton Price, Yovita Hartantri, Bachti Alisjahbana, Rudi Wisaksana, Reinout van Crevel, Andre J. A. M. van der Ven

**Affiliations:** 1 Infectious Disease Research Center, Faculty of Medicine Universitas Padjadjaran, Bandung, Indonesia; 2 Department of Internal Medicine, Radboud University Medical Center, Nijmegen, The Netherlands; 3 Department of Anthropology, Pennsylvania State University, University Park, Pennsylvania, United States of America; 4 Santa Fe Institute, Santa Fe, New Mexico, United States of America; 5 Department of Internal Medicine, Hasan Sadikin General Hospital, Bandung, Indonesia; The Ohio State University, UNITED STATES

## Abstract

**Background:**

Little is known about HIV treatment outcomes in Indonesia, which has one of the most rapidly growing HIV epidemics worldwide.

**Methods:**

We examined possible differences in loss to follow-up (LTFU) and survival between HIV-infected females and males over a 7-year period in an HIV clinic in Bandung, West Java. Data imputation was performed on missing covariates and a multi-state Cox regression was used to investigate the effects of sex and other covariates on patient transitions among four states: (1) clinic enrollment with HIV, (2) initiation/continuation/re-initiation of antiretroviral therapy (ART), (3) LTFU, and (4) death.

**Results:**

We followed 3215 patients (33% females), for a total of 8430 person-years. ART was used by 59% of patients at some point. One-year retention was 73% for females and 77% for males (p = 0.06). One-year survival was 98% for both females and males (p = 0.15). Females experienced a higher relative hazard to transition from HIV to LTFU (adjusted hazard ratio 1.21; 95% confidence interval 1.00–1.45), but this decreased after adjustments for clinical variables (aHR 0.94; 95% CI 0.79–1.11). Similarly, a lower relative hazard in females to transition from ART to death (aHR 0.59; 95% CI 0.35–0.99) decreased after adjustments for demographic variables.

**Conclusion:**

This Indonesian cohort has low ART uptake and poor overall pre- and post-ART retention. Female-male differences in survival and retention were gone after adjusting for clinical and sociodemographic factors such as CD4 count and education level. Efforts should be made to improve retention among patients with lower education.

## Introduction

Indonesia has one of the most rapidly growing HIV epidemics in Southeast Asia with an estimated 690,000 people living with HIV in 2015 [1], mainly in Jakarta, East Java, and West Java [2]. In contrast to the epidemic in sub-Saharan Africa where HIV prevalence rates are higher among women than men [3,4], the early stages of the HIV epidemic in Indonesia (outside Papua) mainly affected male drug injectors [5–7]. In subsequent years, HIV incidence increased among key populations such as men who have sex with men (MSM) and female partners of infected males [8] and improving all aspects of HIV care continuum for these populations is imperative [9]. Little is known about HIV treatment response and retention in care in Indonesia. Previous studies of HIV-infected individuals in Indonesia have focused on survival [10] in association with tuberculosis (TB) and cryptococcal co-infections [11,12], injecting drug use (IDU) [13], and imprisonment [14,15], but there are very limited data on retention in HIV care.

Retention in care, crucial for HIV treatment success [16], remains a major challenge globally. Both worldwide and in Asia about 50% of HIV-infected people received sustained ART in 2016 [17], while Indonesia has a much lower proportion of 14% in 2017 [18]. A recent study found only 76% of HIV-infected key populations who received ART in four cities in Indonesia retained in treatment [19]. In other settings, lower treatment retention has been associated with clinical determinants such as lower CD4 count and TB co-infections [20], health facility and structural-level determinants [21], and social determinants such as lack of a support group [22], IDU [23], imprisonment [24], younger age at ART initiation [25], and lack of occupation or education [26,27]. At the personal level, disease and associated perceived stigma, physical impairments, and general health-seeking behavior influenced retention in care [28].

Existing evidence on the difference of HIV survival and disease progression between males and females is inconsistent [29]. A poorer overall survival in males compared to females has been established in a systematic review and meta analysis of 31 studies [30], with correlations with older age and lower baseline CD4 [31,32]. Females experienced a higher incidence of adverse events [33,34] and treatment discontinuation [35–37], which might play a role in treatment retention. Various measures of lower socioeconomic status have been shown to correlate with poorer treatment outcomes [38,39], including lower pre-ART retention [40]. Studies have shown a lower socioeconomic status among HIV-infected females than males [4,41]. Indonesia experienced a growing proportion of female HIV patients who are usually younger than males at time of diagnosis, with a higher chance of having experienced death of a partner or divorce [42].

Treatment retention can be studied by measuring LTFU, which in most observational studies is treated as a competing risk to survival [43]. Because retention is generally lower before than after ART initiation [44], we decided to investigate LTFU in both pre- and on-ART stages using multistate model [45]. This allowed stage-based effects in disease progression and treatment to be accounted for [46]. This model can be applied to the stages in the HIV care continuum from diagnosis, linkage to care, retention in care, receipt of ART, and viral suppression [47] and has been used to model longitudinal data with unobservable features [48], including in HIV chronicity [49]. Looking at the outcomes of each step in the HIV “care cascade” [50] can inform ‘Test and Treat’ and similar strategies to improve retention. In this study, we compared the rates of LTFU and mortality between females and males in a prospective cohort of HIV patients in Bandung, Indonesia.

## Materials and methods

### Setting and patients

The study population consisted of a cohort of HIV-infected individuals at an HIV outpatient clinic of a provincial referral hospital in Bandung, Indonesia between 2007 and 2014. Patients are enrolled in this clinic for one of two reasons: (1) a newly diagnosed HIV infection or (2) referral from elsewhere with an indication to start or continue ART. As per 2006 WHO recommendation, indication to start ART at the clinic was baseline CD4 <200 cells/mm^3^ or WHO clinical stage III or IV–the baseline CD4 limit was increased to <350 cells/mm^3^ in 2008. Choices of first-line ART offered in the national program are nevirapine (NVP), efavirenz (EFV), zidovudine (ZDV), stavudine (d4T –phased out in 2014), and lamivudine (3TC).

As per routine care, the clinic collects a set of baseline data at the time of enrollment that includes information on health status, HIV transmission risk behavior, and socioeconomic indicators. Regardless of ART status, regular follow-up interviews on risk behavior and reassessment of ART eligibility in patients not yet on ART are planned at 6-month intervals following baseline data collection. Patients receiving ART are expected to come every 30 days to collect their medication, unless they have a special agreement with the attending physician as explained below. Informed consent for research was obtained from patients at the baseline interview and for patients under 18 years of age, with written assent from a parent/guardian.

This study only used routine data, and The Health Research Ethics Committee of the Faculty of Medicine Padjadjaran University in Bandung, Indonesia approved the study. Patient inclusion criteria for this study were: age 15 years or older and non-missing value for date of first contact with clinic. We excluded patients recruited at the narcotics prison, because treatment follow-up for these patients depended on the prison and not the patient.

### Data collection and analysis

Data for this analysis was extracted from the main clinic Microsoft Access database on October 3rd, 2014. Data was recoded and cleaned using Stata version 12 for Mac (Stata Corporation, College Station, TX, USA). Subsequently, reformatting and analysis was conducted in the R programming language [51]. Descriptive statistics for patient characteristics were compared between females and males using a chi-square test for categorical variables with two categories and a Kruskal-Wallis test for categorical variables with more than two categories.

#### Definition and analysis of LTFU

Certain routine practices at the clinic complicate the definition and measurement of LTFU. During follow-up, the clinic does not record the date of the next clinic visit, nor does it actively send reminders to patients of their next appointment. Patients on ART receive medication for exactly 30 days at each visit, unless a special agreement for fewer or more days is made between the patient and the attending physician, in some cases up to 90 days. These arrangements are recorded in the patient record (on paper) and the pharmacy database, but not entered into the clinic’s primary Microsoft Access database. Therefore, a delayed or missed visit for this analysis was estimated from the date of next visit. Tracking of patients with delayed or missed visits is conducted sporadically with phone calls or through outreach workers. Because 180 days of treatment interruption is associated with a higher probability of loss [52] we count patients who experienced such interruptions as LTFU despite possible reengagement into treatment beyond 180 days of interruption.

#### Multi-state analysis

**Fig 1:**
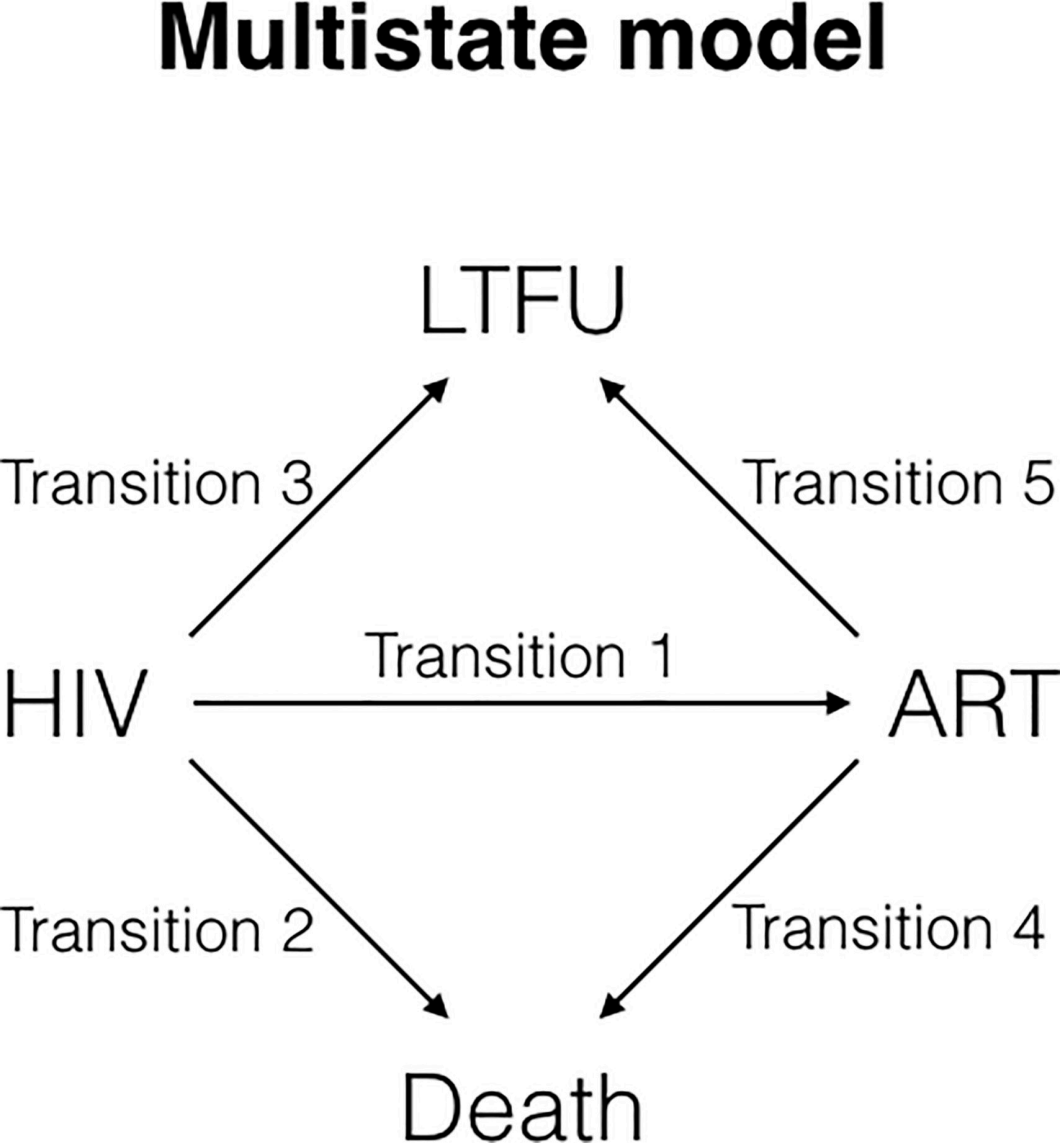
Multi-state model to assess LTFU. State definitions: (1) *HIV state*–started at the earliest recorded date of contact with the clinic for HIV testing, HIV test confirmation, or baseline interview; (2) *ART state*–started on the recorded ART start date or–when this information is missing–the earliest date in the pharmacy record of medicine pick-up. Individuals who received ART on the day they entered the clinic were modeled as starting directly in the ART state; (3) *Death state*–patients entered this state if their status is ‘Dead’ and the date of transition is the recorded date of death; (4) *LTFU state*–patients entered this state if the status is neither ‘Dead’ nor ‘Transferred.’ For patients who transitioned to LTFU from the HIV state, the date of LTFU is calculated as 90 days after the day the patient is expected to come back, i.e. 6 months or 183 days after the last recorded visit, so the date of LTFU is the date of the last recorded visit plus 273. For patients who transitioned to LTFU from the ART state, the date of LTFU is calculated as 60 days after the day the patient is expected to come back, i.e. 30 days after the last recorded visit (the LTFU date is the date of the last recorded visit plus 90 days); in cases where patients experienced multiple interruptions of 180 days or more, the date of LTFU is the date of the first interruption plus 90 days.

Fig 1 summarizes the multi-state model used in this analysis. All patients started in either the *HIV* state (i.e., clinical enrollment with HIV but not on ART) or directly in the ART state. From the *HIV* state, individuals can move to *ART*, *LTFU*, or *Death* states. *LTFU* and *Death* are absorbing states (i.e., final states). There are five possible transitions, each with a distinct hazard function that must be modeled. We used a competing hazards model since more than one transition can occur out of the *HIV* and *ART* states. Confirmation of death was obtained from family or community organization reports or by telephone calls conducted by the clinic [13]. Patients were censored at their end date provided in the clinic’s Access database if their associated last state was ‘Transferred’ or if they had not reached an absorbing state at the administrative censoring date. For the multi-state analysis, we further censored patients who (a) had a final status that was neither ‘Dead’ nor ‘Transferred’ and (b) had a final status date less than 180 days before the administrative censoring date. The latter censoring was conducted in order to count the hazard ratio only of patients whose probability for LTFU was observable in the analysis based on our definition of LTFU.

#### Cox regression

We applied multiple Cox regression to adjust for sociodemographic and clinical variables that influenced treatment outcomes according to the literature [13,26,39,53–57]. All variables were made categorical. The sociodemographic variables considered were age (15–24; 25–39; 40–69), marital status at baseline (single; married; divorced/widowed/separated), home address (Bandung; Greater Bandung; other), education (non-completed basic = finishing only 6 years of schooling or no schooling; basic = finishing 9 years of schooling at elementary and junior secondary schools; secondary = 12 years of schooling up to high school; tertiary = any education beyond high school [58]), and occupation (any type of work; home maker or student; none). The clinical variables considered were ART prior to entry (yes; no), first recorded CD4 count (> 200 cells/mm^3^; <200 cells/mm^3^), Hepatitis C virus (HCV) co-infection (no; yes), TB treatment history (never had TB treatment; ongoing; past treatment–completed; past treatment–incomplete), anemia (no; yes). All the blood sample measurement results taken for this analysis were the earliest one on record. Anemia was included as a proxy measure for overall health [59]. We ran four different models: Model 1 included sex and age as covariates; Model 2 included all sociodemographic variables; Model 3 included sex and clinical variables; and Model 4 included all variables. Regressions were done after imputing missing data with multiple imputation using the R mice (Multivariate Imputation by Chained Equations) package [60]. In all four models we performed 1000 imputations with 20 iterations for each imputation and set the seed to provide reproducible results with a random number generated using www.random.org between 1 and 1,000,000.

## Results

**Fig 2:**
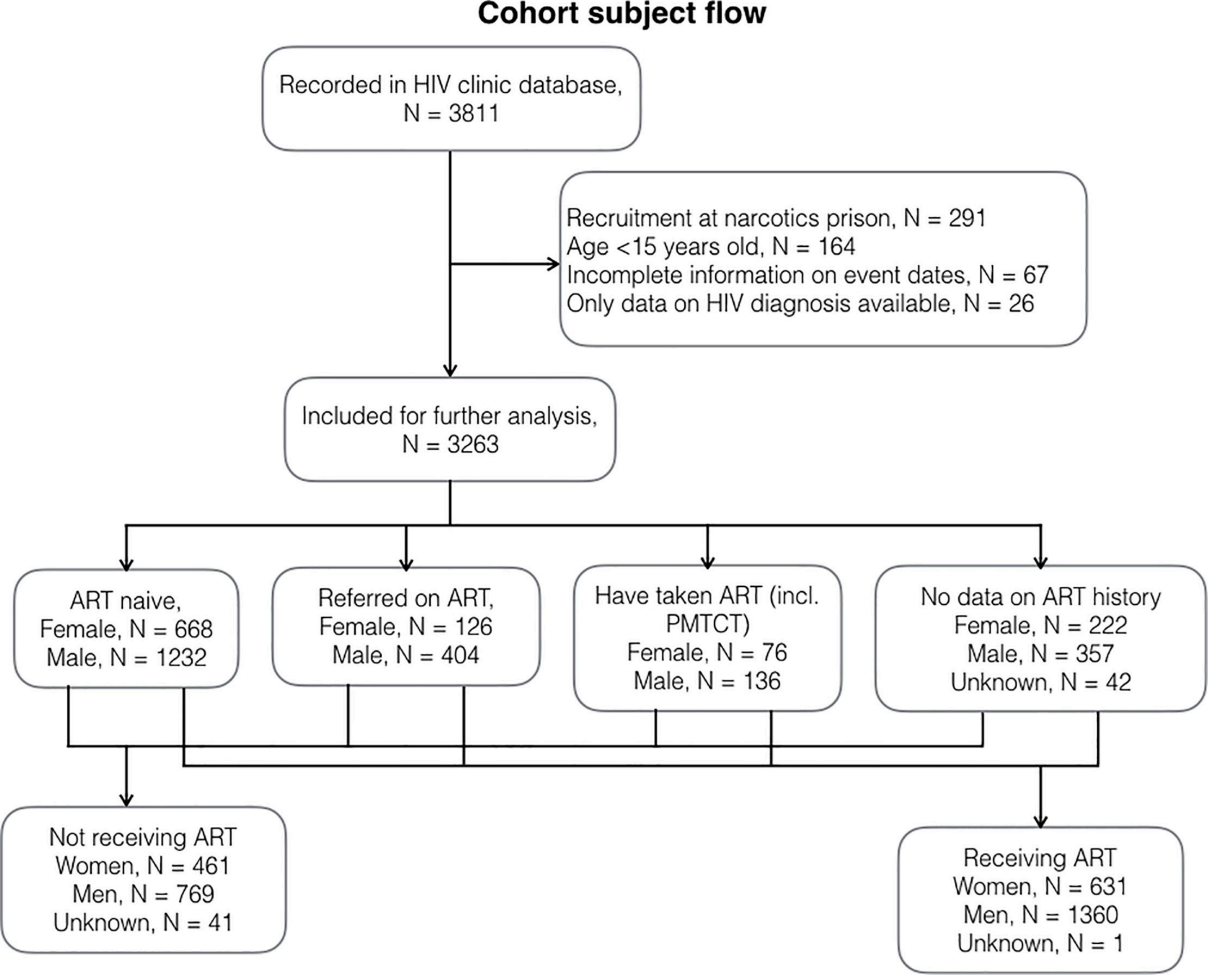
Subject flow.

Between August 1, 2007 and October 3, 2014, 3811 HIV-diagnosed female and male patients were recorded in the database (Fig 2). The following groups were excluded from analysis: patients recruited at the narcotics prison (N = 291); those below 15 years of age (N = 164); those having incomplete information on key event dates (N = 67); and those who were tested for HIV but never had characteristics data collected (N = 26).

### Patient characteristics

Among 3263 patients included, 1900 (58%) presented with newly diagnosed HIV infection, 530 (16%) were referred from other clinics for ART continuation, and 212 (6.5%) were referred with a history of prior ART at some point including for prevention of mother-to-child transmission (PMTCT). We further excluded 48 (1.5%) patients because their censored date was <180 days before the clinic administrative censoring date, and 51 (1.6%) patients because of missing sex data. We included the first CD4 count on record, which was taken within 90 days of first contact with the HIV clinic in 68% of patients who never received ART and was taken before or at ART start in 67% of patients who received ART. In 6% of patients receiving ART, the first recorded CD4 count was taken after ART start, while for the rest of the patients (12%) CD4 count data was not available. Table 1 provides summary statistics of the study population, comparing 1069 females (34%) and 2095 males (66%). Females were slightly younger, more often widowed, divorced, or separated, and more likely to be unemployed. More males had received ART and fewer females had been treated for TB prior to entry. Fewer females had first CD4 count ≤ 200 cells/mm^3^ and hepatitis C co-infection. All differences were consistent when we only compared patients who received ART at the clinic, and all differences were statistically significant (p <0.05).

**Table 1:** Summary of patient characteristics.

	All patients N = 3215		Patients on ART at any time N = 1900	
	Female	Male	Female	Male
**Number (%)**	1069 (34%)	2095 (66%)	613 (32%)	1287 (68%)
**Age category, N (%)**	1035 (34%)	2033 (66%)	612 (32%)	1283 (68%)
15–24	25%	12%	25%	11%
25–39	69%	79%	70%	78%
40–69	6%	10%	5%	10%
**Marital status, N (%)**	904 (33%)	1804 (67%)	583 (32%)	1227 (68%)
Single	11%	45%	9%	44%
Married	62%	47%	63%	49%
Divorced / widowed / separated	27%	8%	28%	7%
**Address, N (%)**	936 (34%)	1855 (66%)	594 (32%)	1234 (68%)
Bandung	54%	61%	58%	64%
Greater Bandung	18%	16%	20%	16%
Other	28%	24%	22%	19%
**Education, N (%)**	887 (33%)	1778 (67%)	577 (32%)	1210 (68%)
Non-completed basic	12%	4%	8%	3%
Basic	16%	9%	13%	8%
Secondary	49%	53%	52%	52%
Tertiary	23%	34%	27%	37%
**Occupation, N (%)**	889 (33%)	1778 (67%)	577 (32%)	1210 (68%)
Any work	36%	76%	36%	77%
Housewife / student	43%	2%	44%	2%
No work	22%	22%	20%	20%
**ART prior to entry, N (%)**	854 (33%)	1750 (67%)	553 (32%)	1187 (68%)
Never had ART	77%	69%	72%	66%
**Baseline CD4 level, N (%)**	1069 (34%)	2095 (66%)	613 (32%)	1287 (68%)
CD4 <200 cells/mm^3^	34%	46%	45%	53%
**Hep C serology, N (%)**	438 (31%)	962 (69%)	288 (31%)	641 (69%)
Anti-HCV positive	24%	76%	26%	78%
**TB treatment history, N (%)**	765 (33%)	1539 (67%)	496 (32%)	1036 (68%)
Never treated for TB	83%	72%	81%	69%
Ongoing treatment	10%	16%	12%	18%
Completed treatment	2%	3%	3%	3%
Incomplete treatment	4%	8%	4%	9%
**Haemoglobin level, N (%)**	780 (33%)	1554 (67%)	502 (32%)	1043 (68%)
Anemia*	52%	46%	53%	45%

Except for home address of patients on ART (*p* = 0.01) and anemia for all patients and patients on ART (*p* = 0.003), all *p*-values were <0.001.

*Haemoglobin <13 g/dl for male and <12 g/dl for female [61]

#### Treatment and follow-up

A total of 3215 patients were included in follow-up. ART was initiated in 1900 (59%) patients. Among 1868 ART-naïve patients, 608 (33%) never started ART, mostly because they either did not return (n = 540) or died (n = 68). Among 696 ART-experienced patients, 133 (19%) did not continue or re-initiate ART. The total follow-up amounted to 8430 person-years and total follow-up on-ART was 4632 person-years. Throughout the study period, 473 patients (15%) were transferred to another facility and thus censored.

#### Patient survival and loss to follow-up, pre- and on ART

**Fig 3:**
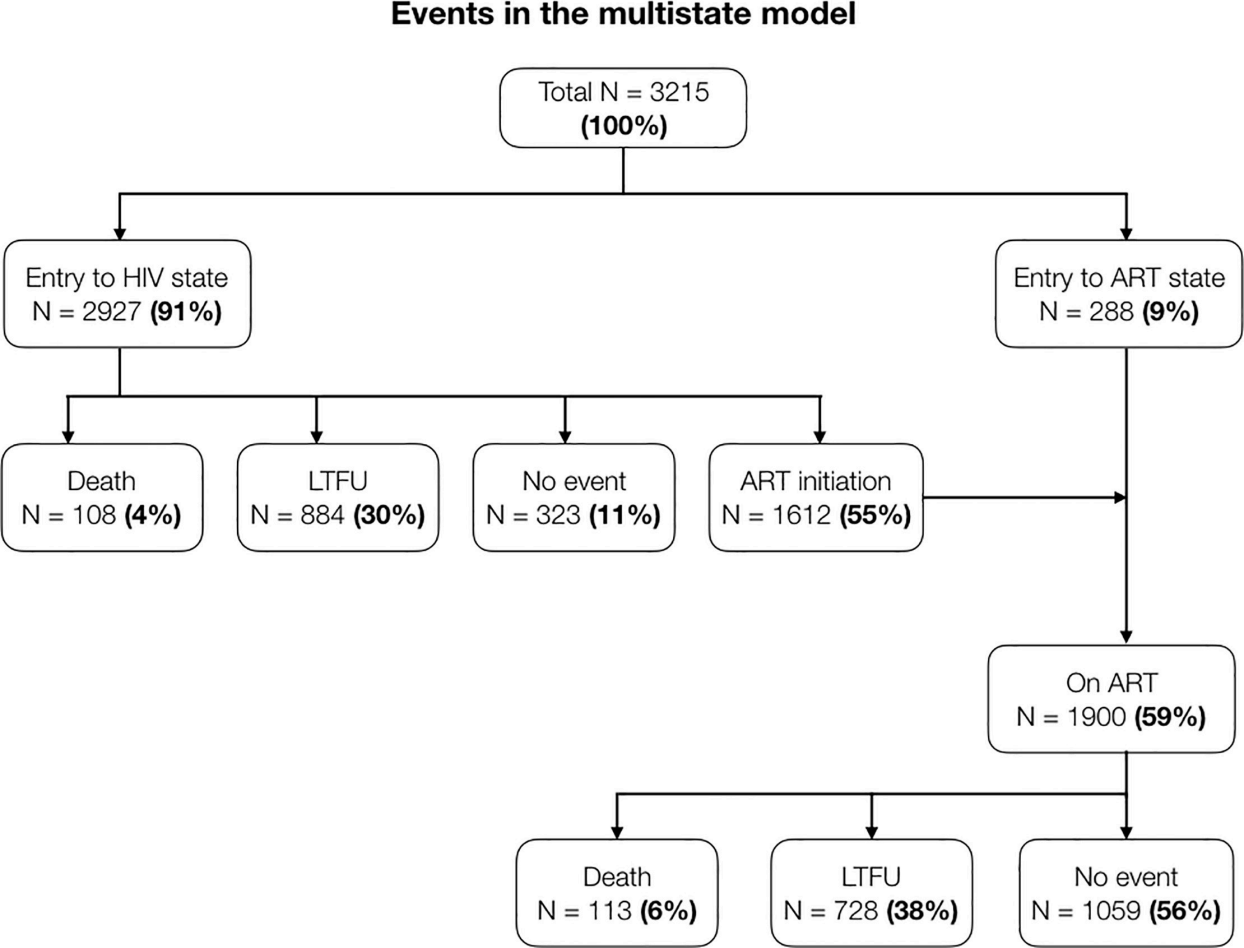
Events in the multistate model.

From a total of 3215 patients entered into the multistate model, 2927 (91%) started in the HIV state while 288 (9%) received ART at entry and started in the ART state (Fig 3). Of all patients on ART (n = 1900), 177 patients (9%) had treatment interruption episodes of 180 days or more (the longest interruption at 1507 days or more than four years); even though they eventually reengaged in care, these patients were treated as LTFU in this analysis. Pre-ART mortality was 4% and pre-ART LTFU was 30%, while in patients receiving ART, mortality was 6% and LTFU 38%. A total of 1059 (56%) patients on ART were retained at the end of the study period.

#### Cox regression

We applied Cox regression to adjust for the associations between sex and the five possible patient transitions between states; i.e., transition from HIV to ART, HIV to Death, HIV to LTFU, ART to Death, and ART to LTFU (Table 2). When adjusting for age, females had a significantly higher hazard ratio to transition from HIV to LTFU (aHR = 1.21; 95%CI = 1.05–1.39). When adjusting for clinical variables, females had a lower hazard ratio to transition from ART to Death (aHR = 0.59; 95%CI = 0.35–0.99) but this effect was gone after also adjusting for demographic variables (aHR = 1.03; 95%CI = 0.89 = 1.21). We found no other significant difference between females and males in other transitions across all models. All outcomes from the models can be found in S1 Table.

**Table 2:** Association between sex and five transitions between states among HIV-infected individuals, giving hazard ratios (with 95% confidence intervals) across four models.

	Model 1		Model 2		Model 3		Model 4	
	HR (95% CI)	p-val	HR (95% CI)	p-val	HR (95% CI)	p-val	HR (95% CI)	p-val
*Transition 1*: *HIV to ART*								
Male	1		1		1		1	
Female	.90 (.81–1.00)	0.05	.92 (.80–1.06)	0.23	1 (.87–1.13)	0.94	1.03 (.89–1.21)	0.68
*Transition 2*: *HIV to Death*								
Male	1		1		1		1	
Female	.86 (.56–1.31)	0.47	0.83 (0.46–1.49)	0.53	.75 (.45–1.23)	0.25	.95 (.48–1.87)	0.88
*Transition 3*: *HIV to LTFU*								
Male	1		1		1		1	
Female	**1.21 (1.05–1.39)**	**0.008**	**1.21 (1.00–1.45)**	**0.05**	.94 (.79–1.11)	0.45	.99 (.80–1.21)	0.89
*Transition 4*: *ART to Death*								
Male	1		1		1		1	
Female	.68 (.43–1.07)	0.10	.68 (.37–1.24)	0.20	**.59 (.35 - .99)**	**0.05**	.67 (.35–1.26)	0.22
*Transition 5*: *ART to LTFU*								
Male	1		1		1		1	
Female	1.08 (.92–1.26)	0.35	1.07 (.87–1.32)	0.52	.95 (.79–1.15)	0.61	.97 (.77–1.22)	0.80

Model 1: adjusted for age

Model 2: adjusted for sociodemographic variables (age, marital status, address, education, and occupation)

Model 3: adjusted for clinical variables (ART history, baseline CD4 count, HCV co-infection, TB treatment history, and anemia)

Model 4: adjusted for sociodemographic and clinical variables

Covariate effects significant at <0.05 are shown in boldface.

## Discussion

There is little published data on HIV treatment outcomes in Indonesia. In this cohort of long term treatment in all HIV-infected patients we found low uptake of ART with many patients failing to start or restart ART and low retention in care (both before and after ART start). The retention and survival patterns were similar in females and males, with females having slightly poorer retention and better survival. ART uptake, retention, and survival with and without ART were influenced by different sociodemographic and clinical variables.

One-year retention in care in this cohort (73% in females and 77% in males) was lower than in the Asia-Pacific region in 2016 (86%) [17]. Poorer pre-ART retention in this study correlated with being older, never having ART prior to entry to the clinic, and anemia. Male sex and lower education were identified as factors influencing pre-ART loss in Mozambique [62], whereas in this study the effect of sex and education diminished after adjusting for clinical variables. A study of HIV-infected key populations in four cities in Indonesia found being diagnosed at a facility that provided both testing and treatment services increased treatment initiation [19]. In this study, lower patient retention after receiving ART was associated with living outside the city and lower education. Analysis of Indonesian MSM and transgender subsample from an Asia Pacific AIDS Positive Network (APN+) study showed an improved retention in care among patients who started ART, had medical insurance, and used the Internet to find HIV-related information [63]. Other studies have associated lower treatment retention with lack of monthly income [64], lower education [54,65], and higher CD4 cell count [65,66]–characteristics that were more common in females than males in this study. Being older and having a higher education were correlated with ART initiation, while unemployed patients less likely to initiate ART. ART initiation was also associated with having a lower CD4 and ongoing treatment for TB. Similar to a study in Rwanda, ART initiation among patients with better clinical presentation seemed to be delayed [67].

Pre-ART survival was poorer for patients who were older, had lower CD4, and had anemia; on-ART survival was poorer for patients living outside the city and who had anemia (S1 Table). The correlation between unemployment and pre-ART survival diminished in the model that adjusted for clinical variables, suggesting a possible interaction between unemployment and poorer clinical presentation. Other studies have linked unemployment with long-term (more than 4 years) mortality during ART [68,69] in settings with higher retention than our cohort. When only adjusting for sex and clinical variables, males had lower on-ART survival, in line with other studies [54,70–72], but adjusting for sociodemographic variables removed this effect in our study. Lower survival, both pre- and on-ART, have also been associated with anemia in this population [73] and in a study in Puerto Rico [74]. Other studies also found an association between lower survival and tuberculosis co-infection [75,76], but we did not find significant associations between TB treatment history and survival in our results (Table 2).

The associations between state transitions and sociodemographic factors in this study are in line with other studies that found effects of low socioeconomic level [40] and migrant status [77] on treatment outcomes. In Europe, the association between education level and ART initiation reflects socioeconomic inequality [78]. Individuals with home addresses outside the city were less likely to receive ART and more likely to experience pre-ART loss. They are typically in the city temporarily for work. A study conducted at the same clinic found family support as a factor that increased retention [79], which migrant workers might lack.

This study has some limitations. We could not account for the probability of dying among LTFU patients due to lack of confirmation of patient deceased status. The effect of low CD4 on pre-ART LTFU supported the hypothesis that some of the patients categorized as LTFU might have died [80]. We included patients who were ART-naïve at entry and those with ART history prior to entry. It is plausible that some patients with ART history have been LTFU prior to entry and they entered this clinic due to symptoms, but our analysis could not account for this possible hidden heterogeneity. There is no standardized definition of LTFU; different definitions yield estimates that vary more than mortality estimates and that are less robust for long-term follow-up [81,82]. We used competing risk analysis to reduce bias of the competing risk of death in analyzing LTFU [83] in a multi-state model investigating each stage of transition [84], and Cox regression to test the multiple factors influencing them [85]. We could not measure treatment failure as an outcome of interest due to lack of CD4 cell plasma HIV-RNA monitoring during treatment [86]. According to the national guideline, patients receiving ART should have a 6-monthly CD4 count and annual HIV-RNA viral load measurements to evaluate treatment response, but while patients can get ART drugs for free, they have to pay for these tests, hence socioeconomic gaps between people with and without lab test data is plausible. We did not account for treatment interruptions, a relatively common occurrence among HIV patients [87] experienced by 97% of patients on ART in this population. Treatment interruption could correlate with treatment failure and retention in care [88,89]. In this cohort we had to censor 473 patients (15%) due to transfer because treatment data between facilities are not linked. Even though censoring transferred patients did not bias mortality estimates in another study [90], a better linkage between various testing and treatment facilities would reduce the need to censor transferred individuals and increase the accuracy of retention estimates [91,92]. We used education level, home address, and occupation–the covariates available in the database as measures of socioeconomic status–but some findings are difficult to interpret. A more in-depth study exploring the relationship between treatment outcomes and socioeconomic status using specific variables would give a better picture [38]. Sex is only one aspect of gender issues influencing health systems but the nature of the study did not allow analysis of gender frameworks and gender power relations in HIV care in this setting [93]. Some baseline patient characteristics in this study have lots of missing data. Patients with missing data on baseline laboratory indicators (CD4 level, hemoglobin, and HCV co-infection) were more likely to not start ART and to be LTFU, and missing information on the history of tuberculosis treatment was significantly related to not starting ART. In this study we used information on TB treatment because information on actual TB diagnosis was not available; hence TB co-infection might be underestimated (an individual with no history of TB treatment may actually have TB). Despite the high occurrence of missing data, we used multiple imputations to yield correctly estimated standard errors and confidence intervals [94]. In our study we also present retention data beyond two-year follow-up, for which there is very little published data [95].

## Conclusions

This study showed a poor pre- and post-ART retention and sex differences that could be explained by sociocultural and clinical characteristics of HIV-patients accessing HIV care and treatment in Indonesia. Efforts should be made to improve retention among patients with lower education levels. However, other aspects of HIV care continuum such as patient retention after treatment initiation remains a challenge. Further studies are needed to investigate the correlation between treatment interruption and treatment failure as well as factors influencing treatment reengagement after an interruption to give insights into ways to improve retention.

## Supporting information

S1 Table Cox regression results of the five state transitions (HIV to ART, HIV to Death, HIV to LTFU, ART to Death, and ART to LTFU). (DOCX)

S1 Datasets Datasets and codes to generate results for descriptive analysis in Table 1 and Cox regression in Table 2 and S1 Table. (ZIP)
