# Supplementary material for: Are there differences in HIV retention in care between female and male patients in Indonesia? A multi-state analysis of a retrospective cohort study
Source: PLoS One. 2019 Jun 25;14(6):e0218781. doi: 10.1371/journal.pone.0218781 (PMC6592601; doi:10.1371/journal.pone.0218781)
Supplement: S1 Table — (DOCX) [file pone.0218781.s001.docx]

**S1 Table. Cox regression results of the five state transitions (HIV to ART, HIV to Death, HIV to LTFU, ART to Death, and ART to LTFU)**

Model 1: gender and age

Model 2: gender, age, and other sociodemographic variables

Model 3: gender and clinical variables

|  | **Model 1** | | | | **Model 2** | | **Model 3** | |
| --- | --- | --- | --- | --- | --- | --- | --- | --- |
|  | HR (95% CI) | | p-val | | HR (95% CI) | p-val | HR (95% CI) | p-val |
| *Transition 1: HIV to ART* | | | | |  |  |  |  |
| **Sociodemographic variables** | | | |  |  |  |  |  |
| **Sex** | |  | |  |  |  |  |  |
| Male | | 1 | |  | 1 |  | 1 |  |
| Female | | 0.90 (0.81-1.00) | | 0.05 | 0.92 (0.80-1.06) | 0.23 | 1 (.87-1.13) | 0.94 |
| **Age** | |  | |  |  |  |  |  |
| 15-24 | | 1 | |  | 1 |  |  |  |
| 25-39 | | **1.17 (1.02 - 1.34)** | | **0.03** | 1.15 (1.00 - 1.25) | 0.23 |  |  |
| 40-69 | | **1.49 (1.20 - 1.84)** | | **<0.001** | **1.50 (1.20 - 1.87)** | **<0.001** |  |  |
| **Marital status** | |  | |  |  |  |  |  |
| Divorced / widowed / separated | |  | |  | 1 |  |  |  |
| Married | |  | |  | 0.98 (0.89 - 1.24) | 0.57 |  |  |
| Single | |  | |  | 0.98 (0.82 - 1.18) | 0.84 |  |  |
| **Address** | |  | |  |  |  |  |  |
| Bandung | |  | |  | 1 |  |  |  |
| Greater Bandung | |  | |  | 1.10 (.95 - 1.27) | 0.19 |  |  |
| Other | |  | |  | **0.71 (.62 - .81)** | **<0.01** |  |  |
| **Education** | |  | |  |  |  |  |  |
| Basic | |  | |  | 1 |  |  |  |
| Non-completed basic | |  | |  | 0.92 (.68 – 1.23) | 0.57 |  |  |
| Secondary | |  | |  | **1.22 (1.02 – 1.47)** | **0.03** |  |  |
| Tertiary | |  | |  | **1.39 (1.14 – 1.69)** | **0.001** |  |  |
| **Occupation** | |  | |  |  |  |  |  |
| Any | |  | |  | 1 |  |  |  |
| Home/student | |  | |  | 1.03 (.86 - 1.24) | 0.73 |  |  |
| None | |  | |  | 0.94 (0.82 - 1.08) | 0.38 |  |  |
| **Clinical variables** | |  | |  |  |  |  |  |
| **ART prior to entry** | |  | |  |  |  |  |  |
| Yes | |  | |  |  |  | 1 |  |
| No | |  | |  |  |  | 1.14 (.99 – 1.30) | 0.06 |
| **Baseline CD4** | |  | |  |  |  |  |  |
| >= 200 | |  | |  |  |  | 1 |  |
| <200 | |  | |  |  |  | **1.89 (1.69 – 2.12)** | **<0.001** |
| **HCV co-infection** | |  | |  |  |  |  |  |
| No | |  | |  |  |  | 1 |  |
| Yes | |  | |  |  |  | 1.05 (.91 - 1.22) | 0.50 |
| **TB treatment history** | |  | |  |  |  |  |  |
| Never | |  | |  |  |  | 1 |  |
| Ongoing | |  | |  |  |  | **1.21 (1.03 – 1.42)** | **0.02** |
| Past-completed | |  | |  |  |  | 0.87 (0.63 – 1.19) | 0.37 |
| Past-incomplete | |  | |  |  |  | 1.03 (0.82 – 1.30) | 0.78 |
| **Anemia*** | |  | |  |  |  |  |  |
| No | |  | |  |  |  | 1 |  |
| Yes | |  | |  |  |  | 0.97 (0.86 – 1.09) | 0.58 |
| *Transition 2: HIV to Deceased* | | | | |  |  |  |  |
| **Sociodemographic variables** | | |  | |  |  |  |  |
| **Sex** | |  |  | |  |  |  |  |
| Male | | 1 |  | | 1 |  | 1 |  |
| Female | | 0.86 (0.56-1.31) | 0.47 | | 0.83 (0.46-1.49) | 0.53 | 0.75 (0.45-1.23) | 0.25 |
| **Age** | |  |  | |  |  |  |  |
| 15-24 | | 1 |  | | 1 |  |  |  |
| 25-39 | | **2.64 (1.36 – 5.14)** | **0.004** | | **2.59 (1.31 – 5.15)** | **0.006** |  |  |
| 40-69 | | **4.90 (2.05 – 11.7)** | **<0.001** | | **5.10 (2.06 – 12.7)** | **<0.001** |  |  |
| **Marital status** | |  |  | |  |  |  |  |
| Divorced / widowed / separated | |  |  | | 1 |  |  |  |
| Married | |  |  | | 1.33 (.67 – 2.67) | 0.42 |  |  |
| Single | |  |  | | 1.07 (.50 – 2.28) | 0.86 |  |  |
| **Address** | |  |  | |  |  |  |  |
| Bandung | |  |  | | 1 |  |  |  |
| Greater Bandung | |  |  | | 0.63 (0.31 - 1.30) | 0.21 |  |  |
| Other | |  |  | | 0.75 (0.45 - 1.25) | 0.27 |  |  |
| **Education** | |  |  | |  |  |  |  |
| Basic | |  |  | | 1 |  |  |  |
| Non-completed basic | |  |  | | 0.34 (0.07 – 1.60) | 0.17 |  |  |
| Secondary | |  |  | | 0.78 (0.42 – 1.49) | 0.46 |  |  |
| Tertiary | |  |  | | 0.70 (0.34 – 1.45) | 0.34 |  |  |
| **Occupation** | |  |  | |  |  |  |  |
| Any | |  |  | | 1 |  |  |  |
| Home/student | |  |  | | 1.23 (.52 – 2.88) | 0.64 |  |  |
| None | |  |  | | **1.95 (1.20 - 3.13)** | **0.006** |  |  |
| **Clinical variables** | |  |  | |  |  |  |  |
| **ART prior to entry** | |  |  | |  |  |  |  |
| Yes | |  |  | |  |  | 1 |  |
| No | |  |  | |  |  | 1.38 (.75 – 2.53) | 0.30 |
| **Baseline CD4** | |  |  | |  |  |  |  |
| >= 200 | |  |  | |  |  | 1 |  |
| <200 | |  |  | |  |  | **2.39 (1.45 – 3.92)** | **<0.001** |
| **HCV co-infection** | |  |  | |  |  |  |  |
| No | |  |  | |  |  | 1 |  |
| Yes | |  |  | |  |  | 0.68 (0.41 – 1.15) | 0.15 |
| **TB treatment history** | |  |  | |  |  |  |  |
| Never | |  |  | |  |  | 1 |  |
| Ongoing | |  |  | |  |  | 1.63 (0.92 – 2.88) | 0.09 |
| Past-completed | |  |  | |  |  | 1.63 (0.60 – 4.42) | 0.33 |
| Past-incomplete | |  |  | |  |  | 1.05 (0.35 – 3.14) | 0.95 |
| **Anemia*** | |  |  | |  |  |  |  |
| No | |  |  | |  |  | 1 |  |
| Yes | |  |  | |  |  | **2.12 (1.23 – 3.66)** | **0.007** |
| *Transition 3: HIV to LTFU* | | | | |  |  |  |  |
| **Sociodemographic variables** | | |  | |  |  |  |  |
| **Sex** | |  |  | |  |  |  |  |
| Male | | 1 |  | | 1 |  | 1 |  |
| Female | | **1.21 (1.05 – 1.39)** | **0.008** | | **1.21 (1.00 – 1.45)** | **0.05** | 0.94 (0.79 – 1.11) | 0.45 |
| **Age** | |  |  | |  |  |  |  |
| 15-24 | | 1 |  | | 1 |  |  |  |
| 25-39 | | **1.44 (1.19 - 1.74)** | **<0.001** | | **1.46 (1.20 - 1.78)** | **<0.001** |  |  |
| 40-69 | | **1.96 (1.45 – 2.66)** | **<0.001** | | **1.88 (1.37 – 2.59)** | **<0.001** |  |  |
| **Marital status** | |  |  | |  |  |  |  |
| Divorced / widowed / separated | |  |  | | 1 |  |  |  |
| Married | |  |  | | 1.09 (0.87 - 1.36) | 0.45 |  |  |
| Single | |  |  | | 1.04 (0.82 - 1.33) | 0.74 |  |  |
| **Address** | |  |  | |  |  |  |  |
| Bandung | |  |  | | 1 |  |  |  |
| Greater Bandung | |  |  | | 1.15 (0.93 - 1.41) | 0.21 |  |  |
| Other | |  |  | | 1.16 (0.99 - 1.37) | 0.07 |  |  |
| **Education** | |  |  | |  |  |  |  |
| Basic | |  |  | | 1 |  |  |  |
| Non-completed basic | |  |  | | 0.96 (0.71 – 1.30) | 0.81 |  |  |
| Secondary | |  |  | | **0.71 (0.58 - 0.89)** | **0.002** |  |  |
| Tertiary | |  |  | | **0.74 (0.58 - 0.94)** | **0.02** |  |  |
| **Occupation** | |  |  | |  |  |  |  |
| Any | |  |  | | 1 |  |  |  |
| Home/student | |  |  | | 0.89 (0.70 - 1.15) | 0.39 |  |  |
| None | |  |  | | 1.11 (0.93 - 1.33) | 0.26 |  |  |
| **Clinical variables** | |  |  | |  |  |  |  |
| **ART prior to entry** | |  |  | |  |  |  |  |
| Yes | |  |  | |  |  | 1 |  |
| No | |  |  | |  |  | **2.08 (1.66 – 2.62)** | **<0.001** |
| **Baseline CD4** | |  |  | |  |  |  |  |
| >= 200 | |  |  | |  |  | 1 |  |
| <200 | |  |  | |  |  | **0.67 (0.56 - 0.80)** | **<0.001** |
| **HCV co-infection** | |  |  | |  |  |  |  |
| No | |  |  | |  |  | 1 |  |
| Yes | |  |  | |  |  | 0.91 (0.75 – 1.10) | 0.32 |
| **TB treatment history** | |  |  | |  |  |  |  |
| Never | |  |  | |  |  | 1 |  |
| Ongoing | |  |  | |  |  | 1.07 (0.81 – 1.42) | 0.62 |
| Past-completed | |  |  | |  |  | 1.03 (0.63 – 1.67) | 0.92 |
| Past-incomplete | |  |  | |  |  | 0.98 (0.68 – 1.42) | 0.91 |
| **Anemia*** | |  |  | |  |  |  |  |
| No | |  |  | |  |  | 1 |  |
| Yes | |  |  | |  |  | **1.26 (1.06 – 1.50)** | **0.01** |
| *Transition 4: ART to Deceased* | | | | |  |  |  |  |
| **Sociodemographic variables** | | |  | |  |  |  |  |
| **Sex** | |  |  | |  |  |  |  |
| Male | | 1 |  | | 1 |  | 1 |  |
| Female | | 0.68 (0.43 - 1.07) | 0.10 | | 0.68 (0.37 - 1.24) | 0.20 | **0.59 (0.35 - 0.99)** | **0.05** |
| **Age** | |  |  | |  |  |  |  |
| 15-24 | | 1 |  | | 1 |  |  |  |
| 25-39 | | 1.05 (.61 – 1.80) | 0.87 | | 1.10 (.63 – 1.90) | 0.74 |  |  |
| 40-69 | | 1.80 (0.83 – 3.91) | 0.14 | | 2.03 (0.91 – 4.54) | 0.08 |  |  |
| **Marital status** | |  |  | |  |  |  |  |
| Divorced / widowed / separated | |  |  | | 1 |  |  |  |
| Married | |  |  | | 0.94 (0.49 - 1.80) | 0.86 |  |  |
| Single | |  |  | | 1.21 (0.61 - 2.44) | 0.59 |  |  |
| **Address** | |  |  | |  |  |  |  |
| Bandung | |  |  | | 1 |  |  |  |
| Greater Bandung | |  |  | | **1.86 (1.19 - 2.91)** | **0.006** |  |  |
| Other | |  |  | | 1.39 (0.83 – 2.32) | 0.21 |  |  |
| **Education** | |  |  | |  |  |  |  |
| Basic | |  |  | | 1 |  |  |  |
| Non-completed basic | |  |  | | 0.91 (0.29 – 2.81) | 0.86 |  |  |
| Secondary | |  |  | | 0.81 (0.44 – 1.48) | 0.49 |  |  |
| Tertiary | |  |  | | 0.57 (0.30 – 1.11) | 0.10 |  |  |
| **Occupation** | |  |  | |  |  |  |  |
| Any | |  |  | | 1 |  |  |  |
| Home/student | |  |  | | 1.04 (0.48 - 2.23) | 0.93 |  |  |
| None | |  |  | | 1.56 (1.00 - 2.43) | 0.05 |  |  |
| **Clinical variables** | |  |  | |  |  |  |  |
| **ART prior to entry** | |  |  | |  |  |  |  |
| Yes | |  |  | |  |  | 1 |  |
| No | |  |  | |  |  | 1.12 (0.68 – 1.83) | 0.66 |
| **Baseline CD4** | |  |  | |  |  |  |  |
| >= 200 | |  |  | |  |  | 1 |  |
| <200 | |  |  | |  |  | 1.58 (0.94 – 2.67) | 0.08 |
| **HCV co-infection** | |  |  | |  |  |  |  |
| No | |  |  | |  |  | 1 |  |
| Yes | |  |  | |  |  | 0.86 (0.53 – 1.40) | 0.54 |
| **TB treatment history** | |  |  | |  |  |  |  |
| Never | |  |  | |  |  | 1 |  |
| Ongoing | |  |  | |  |  | 0.95 (0.57 – 1.56) | 0.82 |
| Past-completed | |  |  | |  |  | 1.01 (0.32 – 3.17) | 0.99 |
| Past-incomplete | |  |  | |  |  | 0.53 (0.17 – 1.65) | 0.28 |
| **Anemia*** | |  |  | |  |  |  |  |
| No | |  |  | |  |  | 1 |  |
| Yes | |  |  | |  |  | **2.27 (1.41 – 3.65)** | **<0.001** |
| *Transition 5: ART to LTFU* | | | | |  |  |  |  |
| **Sociodemographic variables** | | |  | |  |  |  |  |
| **Sex** | |  |  | |  |  |  |  |
| Male | | 1 |  | | 1 |  | 1 |  |
| Female | | 1.08 (0.92 – 1.26) | 0.35 | | 1.07 (0.87 – 1.32) | 0.52 | 0.95 (0.79 – 1.15) | 0.61 |
| **Age** | |  |  | |  |  |  |  |
| 15-24 | | 1 |  | | 1 |  |  |  |
| 25-39 | | 1.07 (0.87 - 1.32) | 0.53 | | 1.06 (0.85 – 1.31) | 0.61 |  |  |
| 40-69 | | 1.25 (0.91 – 1.73) | 0.17 | | 1.20 (0.86 – 1.68) | 0.27 |  |  |
| **Marital status** | |  |  | |  |  |  |  |
| Divorced / widowed / separated | |  |  | | 1 |  |  |  |
| Married | |  |  | | 0.81 (0.64 – 1.02) | 0.07 |  |  |
| Single | |  |  | | 0.98 (0.76 - 1.27) | 0.88 |  |  |
| **Address** | |  |  | |  |  |  |  |
| Bandung | |  |  | | 1 |  |  |  |
| Greater Bandung | |  |  | | 0.90 (0.73 - 1.12) | 0.34 |  |  |
| Other | |  |  | | **1.38 (1.15 - 1.66)** | **<0.001** |  |  |
| **Education** | |  |  | |  |  |  |  |
| Basic | |  |  | | 1 |  |  |  |
| Non-completed basic | |  |  | | **1.91 (1.27 – 2.86)** | **0.002** |  |  |
| Secondary | |  |  | | 1.21 (0.91 – 1.60) | 0.20 |  |  |
| Tertiary | |  |  | | 0.98 (0.72 – 1.32) | 0.87 |  |  |
| **Occupation** | |  |  | |  |  |  |  |
| Any | |  |  | | 1 |  |  |  |
| Home/student | |  |  | | 0.94 (0.73 - 1.22) | 0.66 |  |  |
| None | |  |  | | 1.02 (0.84 - 1.24) | 0.84 |  |  |
| **Clinical variables** | |  |  | |  |  |  |  |
| **ART prior to entry** | |  |  | |  |  |  |  |
| Yes | |  |  | |  |  | 1 |  |
| No | |  |  | |  |  | 0.87 (0.73 – 1.04) | 0.13 |
| **Baseline CD4** | |  |  | |  |  |  |  |
| >= 200 | |  |  | |  |  | 1 |  |
| <200 | |  |  | |  |  | 0.88 (0.75 – 1.04) | 0.14 |
| **HCV co-infection** | |  |  | |  |  |  |  |
| No | |  |  | |  |  | 1 |  |
| Yes | |  |  | |  |  | 0.89 (0.72 – 1.09) | 0.26 |
| **TB treatment history** | |  |  | |  |  |  |  |
| Never | |  |  | |  |  | 1 |  |
| Ongoing | |  |  | |  |  | **0.78 (0.62 - 0.99)** | **0.04** |
| Past-completed | |  |  | |  |  | 0.97 (0.61 – 1.55) | 0.90 |
| Past-incomplete | |  |  | |  |  | 0.73 (0.53 – 1.00) | 0.05 |
| **Anemia*** | |  |  | |  |  |  |  |
| No | |  |  | |  |  | 1 |  |
| Yes | |  |  | |  |  | 1.09 (0.91 – 1.30) | 0.35 |

Covariate effects significant at <0.05 are shown in boldface.

*Haemoglobin <13 g/dl for male and <12 g/dl for female [61]
